# Impact of Oxygen Concentration Delivered via Nasal Cannula on Different Lung Conditions: A Bench Study

**DOI:** 10.3390/healthcare9091235

**Published:** 2021-09-19

**Authors:** Hui-Yun Tseng, Shih-Hsing Yang, Han-Sun Chiang

**Affiliations:** 1 Department of Chemistry, Fu Jen Catholic University, New Taipei City 242062, Taiwan; 090489@gapp.fju.edu.tw; 2 Graduate Institute of Biomedical and Pharmaceutical Science, Fu Jen Catholic University, New Taipei City 242062, Taiwan; 3 Department of Respiratory Therapy, Fu Jen Catholic University, New Taipei City 242062, Taiwan; 075147@mail.fju.edu.tw; 4 Department of Respiratory Therapy, Chang Gung University, Taoyuan 33302, Taiwan

**Keywords:** nasal cannula, oxygen therapy, delivered oxygen concentration, simulation study, normal lung model, restrictive lung model, obstructive lung model

## Abstract

Background: Measuring the fraction of inspired oxygen (FiO_2_) is challenging in spontaneously breathing patients with impaired respiratory mechanics during low-flow nasal cannula. Our study investigates the FiO_2_ with varied tidal volume (V_T_) and respiratory rate (RR) among different lung mechanics and provides equations to estimate the FiO_2_. Methods: Two training and test lungs were used in this study, and the three lung mechanics (normal (R5/C60), restrictive (R20/C80), obstructive (R5/C40)) were designed. Spontaneous breathing with V_T_ (300, 500, and 700 mL) and RR (10, 20, and 30 breaths/min) was simulated. The flow rate of the nasal cannula was set to 1, 3, and 5 L per minute (LPM), and the FiO_2_ was measured at the carina. Results: The lowest and highest FiO_2_ were evident during high (700 mL) and low V_T_ (300 mL), respectively, among normal, restrictive, and obstructive lung models. As RR increases, this decreases the FiO_2_. However, we found that V_T_ and oxygen flow rate are the principal factors influencing measured FiO_2_ by multiple linear regression analysis. Conclusions: Our data suggest that the actual FiO_2_ is never as high in spontaneously breathing patients as that estimated. V_T_ and oxygen flow rate had a substantial impact on the FiO_2_.

## 1. Introduction

Supplemental oxygen is one of the most commonly prescribed treatments to treat spontaneously breathing patients in general units, and for long-term oxygen therapy in the home care setting [1,2,3]. In contemporary clinical practice, 50–84% of patients are exposed to excess oxygen and hyperoxia via various interfaces [1,2,3,4,5]. Recently, a systematic review and meta-analysis provided high-quality evidence that hyperoxia is life-threatening in acutely ill adults with acute myocardial infarction, sepsis, critical illness, stroke, trauma, and cardiac arrest [5].

A nasal cannula is a low-flow oxygen device most widely used in the acute or chronic care setting [1,2,3,4], yet room air dilution is an unavoidable situation [6,7,8,9,10,11]. Rule of thumb suggests that every liter per minute increases the fraction of inspired oxygen (FiO_2_) by approximately 4% for normal rate and depth of breathing [12,13,14]. In sick patients, there is no universal breathing pattern at a different stage; a common pattern is lower tidal volume (V_T_) with an increased respiratory rate (RR) [15,16,17,18,19,20,21,22]. Previous studies reported that estimated FiO_2_ varies from 24% to 33% at 2 L/min (LPM) and from 27% to 50% at 4 LPM [11,23,24,25]. Therefore, the precise FiO_2_ is difficult to measure owing to the variation of the breathing pattern for both V_T_ and RR.

Although the increasing use of continuous monitoring of oxygen saturation by pulse oximetry (SpO_2_) provides an assessment of the adequacy of oxygenation, the risk may not be recognized since SpO_2_ may remain in a satisfactory range because of hyperoxia [12]. The ideal method for determining the FiO_2_ may be to sample tracheal air; however, the invasive procedure has only been performed in small numbers and may lead to changes in patterns [26].

No systemic attempt has been made to estimate the FiO_2_ and its likely variability in patients with obstructive and restrictive lung diseases through a nasal cannula. Therefore, we conducted this bench study to investigate the performance of a nasal cannula in a manikin head-test lung-ventilator system to simulate a spontaneous breathing pattern in normal, restrictive, and obstructive lung models. Our aim was to describe the effects of various V_T_ and RR on the measured FiO_2_ at the carina and provide equations to estimate the FiO_2_ during standard nasal cannula oxygen therapy.

## 2. Materials and Methods

### 2.1. The Lung Model

**Figure 1:**
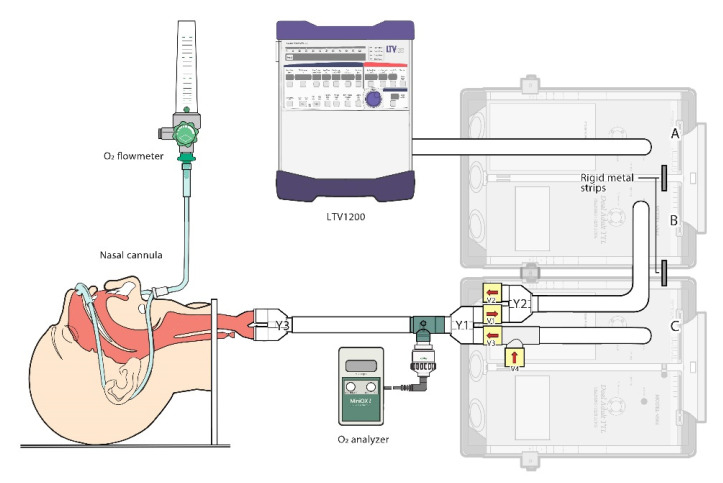
Experimental apparatus of the test lung model. (**A**) driving bellow; (**B**) ventilation bellow; (**C**) expiratory gas modification bellow was constructed.

An experimental apparatus was constructed as shown in Figure 1. Spontaneous breathing was generated with a mechanical ventilator (LTV 1200, CareFusion, San Diego, CA, USA) and two sets of two compartments training and test lung (TTL, Model 5600i, Michigan Instruments, Grand Rapids, Michigan) [26]. We linked the two TTLs with two rigid metal strap manually for three bellows. The driving bellow (A) and ventilation (B) bellow were used to achieve spontaneous breathing simulation, while the expiratory gas modification bellow (C) was used to generate the expiration gas flow. The driving bellow was connected to an LTV1200 ventilator, which allows for changes in RR and V_T_. As the driving bellow expands, a reciprocal negative pressure is produced in the ventilation bellow. This acts as “inspiration”, through the in-line one-way valve 1. Gas is thus “inhaled” into the ventilation bellow, which is connected to a human-like anatomy airway model (Laerdal Airway Management Trainer 25 00 00) with oxygen therapy applied via a nasal cannula (VADI Medical Technology, Taoyuan, Taiwan), and an oxygen analyzer (MiniOX I; MSA Medical, Gurnee, IL, USA) was connected to the bronchi for the measurement of FiO_2_. During expiration, the air was exhaled from the ventilation bellow through the one-way valve 2. To simulate the expired gas in the trachea, room air was inhaled through one-way valve 4 into the expiratory gas modification bellow during inspiration period. Hereafter, the air was exhaled to the anatomic dead space in the airway trainer through the one-way valve 3 during the expiration period.

### 2.2. Testing Protocol

This experiment included three levels of resistance (R in L/s/cmH_2_O) and compliance (C in mL/cmH_2_O) of TTL to represent the lung mechanics of normal, obstructive, and restrictive lung diseases, as suggested by the manufacturer and previous studies [27,28,29,30]. In the normal lung model, R5/C60, R20/C80, and R5/C40 were represented as obstructive and restrictive lung diseases, respectively. The protocols were performed with a wide-ranging change in the ventilatory pattern by the LTV1200 ventilator (volume control mode; low V_T_: 300, normal V_T_: 500, and high V_T_: 700 mL; low RR:10, normal RR: 20, and high RR: 30 breaths/min); oxygen flow rates of 1, 3, and 5 LPM were set for the nasal cannula.

### 2.3. Variables and Measurements

Our primary outcome was determining the FiO_2_. Before beginning the experiment, the oxygen analyzer was calibrated according to the manufacturer’s instructions using 50 psi wall gas source of oxygen and air. Oxygen concentration measurements were obtained at equilibrium, which was assumed to occur when the reading was steady over the 3 min period. In each experiment, the FiO_2_ was recorded for 10 breaths and triplicated for each experimental condition. In addition, to verify the pressure generated at the beginning of inhalation, we measured the pressure change at the carina to represent the inspiratory driving pressure during various lung mechanics.

### 2.4. Statistics

Means and standard deviations (SDs) were calculated within repeated sampling. The small SD of each condition confirmed the stability and reproducibility of the measurements in our simulated model. A one-way ANOVA with a post hoc Tukey’s was performed to test between-group differences. In addition, to test the predictive factors associated with the FiO_2_, multiple linear regression analysis was performed to determine whether V_T_, RR, and oxygen flow rate were predictors. Statistical analysis was performed using SPSS 22.0 software (SPSS Inc., Chicago, IL, USA). A *p* value of <0.05 was considered statistically significant.

## 3. Results

### 3.1. The Lowest Inspiratory Driving Pressure Obtained through the Obstructive Lung Model

**Figure 2:**
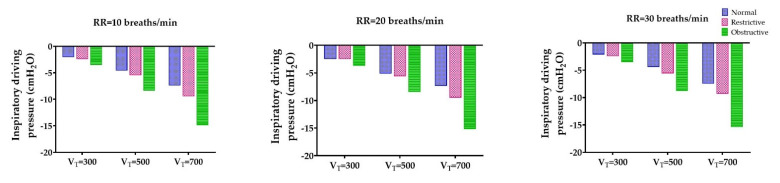
Effect of tidal volume (V_T_) on inspiratory driving pressure.

We observed that the inspiratory driving pressure generated in the obstructive lung model was greater than that in the normal and restrictive lung disease models, regardless of RR and V_T_ settings in our experiment (Figure 2). However, the trend of decreased inspiratory driving pressure was more profound in the high V_T_ (700 mL) than in the low (300 mL) and normal (500 mL). For example, at the same RR of 30 breaths/min, the inspiratory driving pressure decreased from −2.1 to −7.4 cmH_2_O, −2.4 to −9.3 cmH_2_O, and −3.5 to −15.4 cmH_2_O in the normal, restrictive, and obstructive lung mechanics settings, respectively, for V_T_ of 300, 500, and 700 mL.

### 3.2. Decreased Fraction of Inspired Oxygen as Tidal Volumes Increased

**Figure 3:**
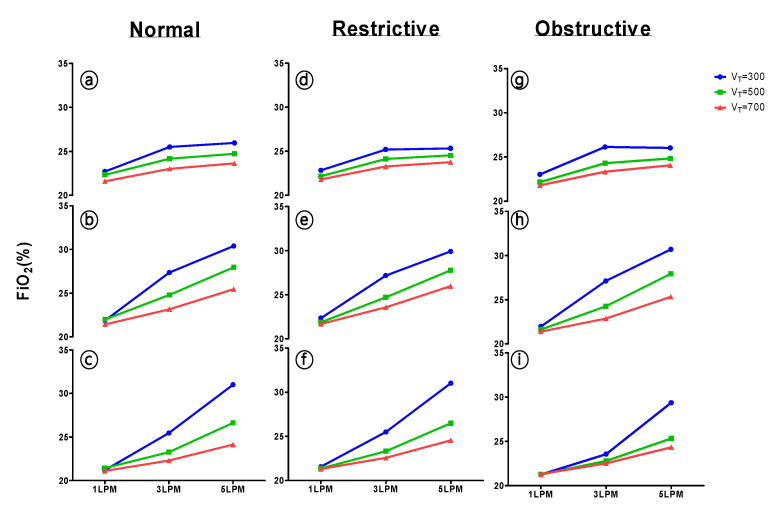
Effect of tidal volume (V_T_) on FiO_2_. (**a**,**d**,**g**) RR: 10 breaths/min.; (**b**,**e**,**h**) RR: 20 breaths/min.; (**c**,**f**,**i**) RR: 30 breaths/min.

In normal, restrictive, and obstructive lung conditions, as V_T_ increased, the measured FiO_2_ showed a statistically significant difference at all oxygen flow rates (*p* < 0.05) during low, normal, and high RR settings (Figure 3). In normal lung mechanics, at low RR (10 breaths/min) with 1 LPM oxygen flow delivery, the measured FiO_2_ was 1.116 times greater at low V_T_ (300 mL) than at normal V_T_ (500 mL); conversely, it was 0.966 times greater at high V_T_ than at normal V_T_. Moreover, when the obstructive lung model set at V_T_ 300, 500, and 700 mL and RR 20 breaths/min, with 1 LPM oxygen delivery, the FiO_2_ was 21.97 ± 0.31%, 21.63 ± 0.12%, and 21.40 ± 0.1%. With 3LPM, it was found that the increase of TV results in the decrease of the FiO_2_, 27.13 ± 0.31%, 24.27 ± 0.06%, and 22.87 ± 0.15% with V_T_ of 300, 500, and 700 mL, respectively. A s result was identified with 5 LPM. When V_T_ increased from 300 to 700 mL, the FiO_2_ decreased from 30.70 ± 0.61% to 27.93 ± 0.12%. The restrictive lung model showed the same trend. Consequently, at the same RR, a smaller FiO_2_ was recorded when a larger V_T_ was present.

### 3.3. Effect of Fraction of Inspired Oxygen by the Change of Respiratory Rate

**Figure 4:**
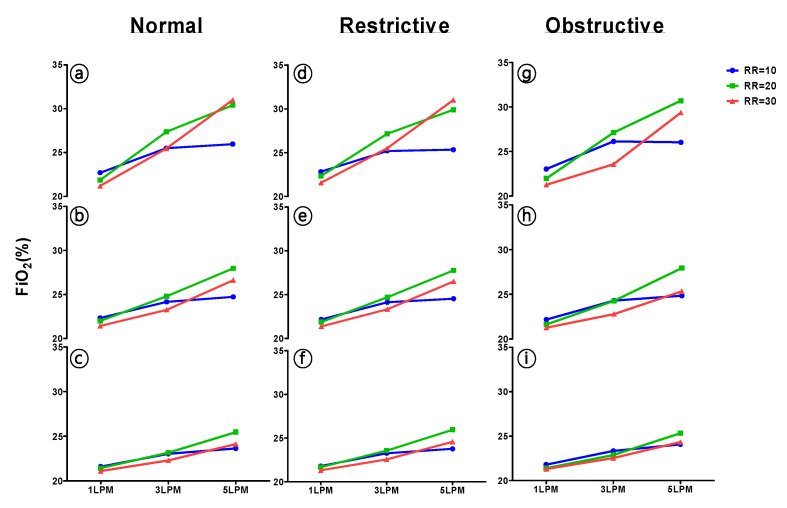
Effect of respiratory rate (RR) on FiO_2_. (**a**,**d**,**g**) V_T_: 300 mL.; (**b**,**e**,**h**) V_T_: 500 mL.; (**c**,**f**,**i**) V_T_: 700 mL.

We observed a different measured FiO_2_ with various RRs (Figure 4). At an oxygen delivery rate of 3 LPM in V_T_ 500 mL with the normal lung model, the FiO_2_ decreased by 0.63% between 20 and 10 breaths/min (*p* < 0.001), by 1.53% between 20 and 30 breaths/min (*p* < 0.001), and by 0.90% between 10 and 30 breaths/min (*p* < 0.001). In addition, we found that under low, normal, and high RR settings, a high V_T_ (700 mL) recorded the smallest difference compared with normal (500 mL) and low V_T_ (300 mL). As in the normal lung model with 3 LPM oxygen flow rate, the difference between FiO_2_ measurements from RR 10 to RR 20 breaths/min was 0.133% in high V_T_, and 0.633% and 1.867% in normal and low V_T_, respectively.

### 3.4. Tidal Volume and Oxygen Flow Rate Were Impact Factors on Oxygen Delivery

According to the above results, FiO_2_ measurements were affected by V_T_, RR, and oxygen flow rate. Therefore, we tested their relationship using multiple linear regression analysis (Table 1). In normal, restrictive, and obstructive lung models, we found no obvious effect of RR on measured FiO_2_, but these appeared statistically significantly affected by the V_T_ and oxygen flow rate. The linear model produced Equation (1) for the normal lung, Equation (2) for the restrictive lung, and Equation (3) for the obstructive lung, as follows:

(1) $${\mathrm{FiO}}_{2}=24.181-0.447\times V_{T}\left(\mathrm{liter}\right)+0.753\times O_{2}\;\mathrm{flow}\;\mathrm{rate}$$

(2) $${\mathrm{FiO}}_{2}=23.857-0.405\times V_{T}\left(\mathrm{liter}\right)+0.768\times O_{2}\;\mathrm{flow}\;\mathrm{rate}$$

(3) $${\mathrm{FiO}}_{2}=23.680-0.406\times V_{T}\left(\mathrm{liter}\right)+0.769\times O_{2}\;\mathrm{flow}\;\mathrm{rate}$$

## 4. Discussion

Our experiment demonstrated that the variation of oxygen flow rate, V_T,_ and RR with a low-flow nasal cannula influenced the delivery of oxygen concentrations in the normal, restrictive, and obstructive lung models. Our findings also showed that despite the different RR, a greater increase in measured FiO_2_ was seen in low V_T_ (300 mL) than in normal (500 mL) and high (700 mL). Compared with RR 10 breaths/min and RR 30 breaths/min, FiO_2_ measured by RR 20 breaths/min had a higher value. In addition, we developed equations for low-flow nasal cannula oxygen therapy for clinicians’ reference.

Different research designs and monitoring locations result in a wide range of FiO_2_ measured. During oxygen therapy with nasal cannula, the rule of thumb is that for patients with a normal rate and depth of breathing, each 1 LPM of oxygen supplied increases FiO_2_ by approximately 4% [1,2,3]; therefore, the expected delivery is a FiO_2_ of 24–44%. However, if the patient’s RR approaches or exceeds 20 breaths/min, FiO_2_ will likely be well below the estimates, and the delivered FiO_2_ will only increase by approximately 2.5% for each 1 LPM above the ambient oxygen level [1,31]. Wagstaff et al. reported that effective inspired oxygen concentration with nasal cannula was 69.67% (RR 10 breaths/min) and 48% (RR 30 breaths/min) at 2 LPM, and 81.22% (RR 10 breaths/min) and 54.67% (RR 30 breaths/min) at 6 LPM during a V_T_ of 500 mL via sampling as far down the airway [32]. In those studies, the FiO_2_ was measured by an oxygen analyzer placed in the lower or distal airway, for example, using an oxygen analyzer port of the TTL bellow. Chikata et al. measured the FiO_2_ of inspired gas downstream of the external nares and found that FiO_2_ with V_T_ of 300, 500, and 700 mL was 0.37 ± 0.01, 0.32 ± 0, and 0.29 ± 0 at 2 LPM; 0.45 ± 0.01, 0.39 ± 0.01, and 0.34 ± 0 at 4 LPM; and 0.58 ± 0.01, 0.45 ± 0.01, and 0.40 ± 0 at 6 LPM [33].

Unlike previous models that measured the FiO_2_ in the lower airway, we measured the FiO_2_ at the carina. Lower measured FiO_2_ than estimated were found at all settings, which might have been due to differences in monitoring sites and the experimental design. By contrast, Gibson et al. reported that in healthy subjects with a percutaneously placed tracheal sensing catheter, the highest absolute inspired tracheal oxygen concentration with a nasal cannula was 23.6% at 3 LPM and 25.4% at 5 LPM during normal breathing (V_T_: 690 mL; RR: 17 breaths/min; minute ventilation (MV): 11 L/min; and peak inspiratory flow rate (PIFR): 37 L/min), 22.4% at 3 LPM and 23.8% at 5 LPM during quiet breathing (V_T_: 400 mL; RR: 16 breaths/min; MV: 6.4 L/min; and PIFR: 21 L/min), and 22.7% at 3 LPM and 25.2% at 5 LPM during hyperventilation (V_T_: 1400 mL; RR: 14 breaths/min; MV: 19.5 L/min; and PIFR: 63 L/min) [11]. Caille et al. used a cadaveric preparation to preserve upper airway patency; the measured FiO_2_ was 22.8 ± 0.6% and 31.2 ± 3.2% at oxygen flow rates of 1 and 3 LPM with a nasal cannula [34]. In another report, in patients with 97% oxygen supply via nasal cannula, the effective FiO_2_ was 22.8 ± 0.1%, 27.6 ± 0.5%, and 31.8 ± 0.5% during oxygen supply at 1, 3, and 5 LPM based on trachea sampling, respectively [33]. Our findings concur reasonably well with those data, but contrast with a previously published formula. However, FiO_2_ is difficult to measure in spontaneously breathing patients and is influenced by the heterogeneous ventilatory conditions observed in clinical practice. Furthermore, the oxygen flow rates higher than 4 LPM significantly disturbs a patient, causes irritation, and dries nasal mucosae. If required, an oxygen mask may be applied.

It is commonly believed that the effectiveness of low-flow oxygen supply systems is affected by the patient’s V_T_ and inspiratory flow rate [1,2,3]. This conclusion is consistent with our results. A study by Chikata et al. reported that statistically significant changes in V_T_ affected measured FiO_2_ at all flow levels: at 2 LPM and V_T_ of 300, 500, and 700 mL, FiO_2_ was 0.37 ± 0.01, 0.32 ± 0 and 0.29 ± 0, respectively; and at 4 LPM, FiO_2_ was 0.45 ± 0.01, 0.39 ± 0.01, and 0.34 ± 0, respectively [33]. Various authors have reported decreasing FiO_2_ values when increasing MV and RR [9]. Gibson et al. reported that the air dilution effect of hyperventilation was evidenced by lower tracheal fractions compared with those of normal or quiet breathing [11]. Our data are consistent with previous studies demonstrating that changes in V_T_ affect the measured oxygen concentration. This suggests that as V_T_ increased (V_T_ 700 mL) with a fixed RR in the normal, restrictive, and obstructive lung models, larger inspiratory flow rates were generated, more room air was inspired to the dilution of the oxygen concentration, and thus lower measurements of FiO_2_ were found.

Previous studies indicated that the FiO_2_ is affected by the RR, the inspiratory time, the presence of a reservoir from which entrainment occurs, and the presence of an expiratory pause [10,27]. The increase in FiO_2_ with increasing oxygen flow rates through an Ncan has been demonstrated in a mannequin model [29,32] and humans [7,11,24,35]. The study by Gibson et al., which used a nasal cannula for the administration of low-flow oxygen, resulted in only slight increases in tracheal oxygen concentration (23.6% at 3 LPM and 25.4% at 5 LPM). The air dilutional effect of hyperventilation was evidenced by lower tracheal fractions compared with those of normal or quiet breathing [11]. Our findings concur reasonably well with those data, but contrast with a published formula that would predict the FiO_2_ to be 32% at 3 LPM and 40% at 5 LPM. Zhou and Chatburn reported that inspiratory time and anatomic reservoir (AR) are key factors during nasal cannula therapy. As the frequency increases (due to either exercise or disease), the inspiratory time decreases from one second to 0.5 s, which in turn decreases FiO_2_ from 34% to 32%, referred to as the frequency effect. Patients with COPD (chronic obstruction pulmonary disease) often have non-zero expiratory flows when the next inspiratory effort is made (i.e., they have alveolar gas-trapping or long expiratory time); thus, end-expiratory flow could prohibit oxygen accumulating in the AR, in this case, decreasing FiO_2_ from 34% to 26% [27]. This refers to the reservoir effect. Sim [10] and O’Reilly [36] also demonstrated that decreased FiO_2_ was due to prevent AR during a stress breathing pattern in a mannequin model. In our experiments with different lung mechanical models (normal, restrictive, and obstructive), we also found that the breathing frequency increases, which in turn decreases FiO_2_; this suggests that frequency and prohibited AR effects should be considered during oxygen administration. A relatively low breathing rate resulted in more stability in measured FiO_2_ during the 3 and 5 LPM oxygen supply; therefore, we speculate that there is sufficient time to fill the oxygen flush in the AR during the zero expiratory flow and before the next inspiratory effort is made.

Our results show that the FiO_2_ measured at the carina by RR 20 breaths/min had a higher value compared with FiO_2_ measured by RR 10 and 30 breaths/min. This fascinating phenomenon of FiO_2_ variation at the conduction airway from nasal to carina during oxygen delivery is not easily observed in the clinic.

In our human-like anatomy model, while the breathing pattern with RR 10 breaths/min exhibited an FiO_2_ plateau on setting the oxygen flow rate from 3 to 5 LPM, presumably the longer expiratory time may have provided more time to mix oxygen and fresh air from the oral cavity, resulting in an increased FiO_2_ that was not obvious. The finding is similar to a clinical study of Nugent et al., which indicated that the RR factor can affect the FiO_2_ [36]. However, compared with normal (20 breaths/min) and higher (30 breaths/min) breathing rates, we demonstrated that an increased RR decreases the FiO_2_, which is related to insufficient oxygen filling time and reduces oxygen reservation in the airway. Thus, any variation in AR affects the composition of the next inspiration. In addition, a more decreased difference of measured FiO_2_ during high V_T_ (700 mL) than during normal V_T_ (500 mL) and low V_T_ (300 mL) at different breathing rates suggests that the high inspiratory flow rate results in more room air dilution. This resembles the results of Wagstaff [32] and Sim [10], wherein a higher RR and shorter expiratory time led to the air dilution effect of FiO_2_ in the airway anatomic dead space and reduced oxygen reserves. The RR 20 breaths/min is a valuable reference during the use of nasal cannula, which suggests that RR 20 breaths/min provides an *opportune* time to mix inspired room air and oxygen in the AR space.

According to our calculations, the prediction formulas are suited to adult patients with normal lungs and those with restrictive or obstructive lung disease. Our study showed that V_T_ and oxygen flow rate affect the measured FiO_2_ rather than RR. Therefore, we proposed equations for future clinical practice with nasal cannula oxygen therapy during spontaneous breathing. These equations are based on different lung mechanics, V_T_, and oxygen flow rates, and they will provide clinicians with a reference when using nasal cannula among patients with normal lungs and those with restrictive and obstructive lung disease.

Our results provide a salutary reminder about the variability of the FiO_2_ delivered via nasal cannula. When using the nasal cannula, clinicians must carefully consider the alveolar PO_2_ and the physiological shunt, based on the alveolar-arterial oxygen difference to decrease the risk of hypoxemia.

This study had several limitations. First, regarding our research design, we controlled the V_T_, RR, and inspiratory time using a simulating ventilator that differed from natural conditions. In addition, we did not consider the factors of open or closed mouth breathing, although the oxygen concentration was influenced by the reservoir space (oral cavity), and the rebreathing phenomenon of CO_2_ in the low oxygen supply flow. Additionally, the necessity of humidified oxygen for patients should take into consideration the effect of the oxygen concentration of water vapor.

Second, a previous study demonstrated that a larger inspiratory flow rate consequently lowered the measured FiO_2_ because of the higher amount of inspired room air dilution [11]. However, we did not measure the inspiratory peak flow in our experiment, which is a limitation of our research. Third, measurement of FiO_2_ was affected by the shunt and dead space during ventilation of the restrictive and obstructive lung disease models. Obviously, a mannequin model cannot simulate this complexity, which is a limitation of our study.

## 5. Conclusions

Since room air dilution was an unavoidable situation in low-flow oxygen therapy and the apparatus dead space varied between devices, it was necessary to estimate the FiO_2_ value to assure one device brought up the oxygen concentration. The data obtained suggest that the actual inspired percentage of oxygen reaching the trachea is never as high in spontaneously breathing patients as that estimated. According to our observations, the V_T_ and oxygen flow rate had a substantial impact on the FiO_2_ and can therefore lead to over or under oxygenation without careful monitoring. Our experiment proposed a prediction formula regarding various lung mechanics and respiratory patterns that considers oxygen flow rate and V_T_ for clinical application.

Although our result provides a salutary reminder about the variability of the FiO_2_ delivered via nasal cannula, when using the nasal cannula, clinicians must carefully consider the alveolar PO_2_ and the physiological shunt, based on the alveolar-arterial oxygen difference, to decrease the risk of the hypoxemia.

## Figures and Tables

**Table 1:** Multiple Linear Regression Analysis Predicting the measured FiO_2_.

Models	Variables		Standardized β Coefficients	*t* Value ^b^	*p* Value	Collinearity Statistics	
						Tolerance	VIF
Normal	Constant	24.181		44.682			
	O_2_ flow rate		0.753	13.758 **	<0.001	1.000	1.000
	V_T_		−0.447	−8.164 **	<0.001	1.000	1.000
	RR		0.037	0.503	0.161	1.000	1.000
Restrictive	Constant	23.857		45.642			
	O_2_ flow rate		0.768	13.665 **	<0.001	1.000	1.000
	V_T_		−0.405	−7.208 **	<0.001	1.000	1.000
	RR		0.085	1.182	0.241	1.000	1.000
Obstructive	Constant	23.680		46.112			
	O_2_ flow rate		0.769	13.790 **	<0.001	1.000	1.000
	V_T_		−0.406	−7.282 **	<0.001	1.000	1.000
	RR			−1.000	0.320	1.000	1.000

^b^ Calculated from t distribution. ** *p* < 0.001.

## Data Availability

Not applicable.

## References

[1] Hess D.R., Macintyre N.R., Galvin W.F. (2016). Respiratory Care: Principles and Practice.

[2] Kacmarek R.M., Stoller J.K., Heuer A.J. (2016). Egan’s Fundamentals of Respiratory Care.

[3] Cairo J.M. (2013). Mosby’s Respiratory Care Equipment.

[4] O’Driscoll B.R., Howard L.S., Earis J., Mak V. (2017). BTS guideline for oxygen use in adults in healthcare and emergency settings. Thorax.

[5] Chu D.K., Kim L.H.-Y., Young P.J., Zamiri N., Almenawer S.A., Jaeschke R., Szczeklik W., Schünemann H.J., Neary J.D., Alhazzani W. (2018). Mortality and morbidity in acutely ill adults treated with liberal versus conservative oxygen therapy (IOTA): A systematic review and meta-analysis. Lancet.

[6] Leigh J.M. (1973). Variation in performance of oxygen therapy devices. Towards the rational employment of ‘The dephlogisticated air described by Priestley’. Ann. R. Coll. Surg. Engl..

[7] Bazuaye E.A., Stone T.N., Corris P.A., Gibson G.J. (1992). Variability of inspired oxygen concentration with nasal cannulas. Thorax.

[8] Davies R.J., Hopkin J.M. (1992). Variability of inspired oxygen concentration with nasal cannulas. Thorax.

[9] Ooi R., Joshi P., Soni N. (1992). An evaluation of oxygen delivery using nasal prongs. Anaesthesia.

[10] Sim M.A.B., Dean P., Kinsella J., Black R., Carter R., Hughes M. (2008). Performance of oxygen delivery devices when the breathing pattern of respiratory failure is simulated*. Anaesthesia.

[11] Gibson R.L., Comer P.B., Beckham R.W., McGraw C.P. (1976). Actual Tracheal Oxygen Concentrations with Commonly Used Oxygen Equipment. Anesthesiology.

[12] McDonald C.F. (2014). Low-flow oxygen: How much is your patient really getting?. Respirology.

[13] Yamamoto N., Miyashita T., Takaki S., Goto T. (2015). Effects of Breathing Pattern on Oxygen Delivery Via a Nasal or Pharyngeal Cannula. Respir. Care.

[14] Nishimura M. (2016). High-Flow Nasal Cannula Oxygen Therapy in Adults: Physiological Benefits, Indication, Clinical Benefits, and Adverse Effects. Respir. Care.

[15] Ware L.B., Matthay M.A. (2000). The acute respiratory distress syndrome. N. Engl. J. Med..

[16] Nava S., Larovere M.T., Fanfulla F., Navalesi P., Delmastro M., Mortara A. (2003). Orthopnea and inspiratory effort in chronic heart failure patients. Respir. Med..

[17] Poletti V., Tomassetti S., Ravaglia C. (2018). Idiopathic pulmonary fibrosis. N. Engl. J. Med..

[18] Branson R.D. (2018). Oxygen Therapy in COPD. Respir. Care.

[19] Pavlov N., Haynes A.G., Stucki A., Jüni P., Ott S.R. (2018). Long-term oxygen therapy in COPD patients: Population-based cohort study on mortality. Int. J. Chronic Obstr. Pulm. Dis..

[20] Iyer V.N. (2018). Low-dose oxygen therapy in copd patients: Are there any radiation-like risks?. Curr. Opin. Pulm. Med..

[21] Inwald D., Roland M., Kuitert L., McKenzie S.A., Petros A. (2001). Oxygen treatment for acute severe asthma. BMJ.

[22] Tokuda Y., Miyagi S. (2001). Oxygen treatment for acute severe asthma. Home oxygenation would be more effective. BMJ.

[23] Schacter E.N., Littner M.R., Luddy P., Beck G.J. (1980). Monitoring of oxygen delivery systems in clinical practice. Crit. Care Med..

[24] Waldau T., Larsen V.H., Bonde J. (1998). Evaluation of five oxygen delivery devices in spontaneously breathing subjects by oxygraphy. Anaesthesia.

[25] Wettstein R.B., Shelledy D.C., Peters J.I. (2005). Delivered oxygen concentrations using low-flow and high-flow nasal cannulas. Respir. Care.

[26] Duprez F., Mashayekhi S., Cuvelier G., Legrand A., Reychler G. (2018). A New Formula for Predicting the Fraction of Delivered Oxygen During Low-Flow Oxygen Therapy. Respir. Care.

[27] Zhou S., Chatburn R.L. (2014). Effect of the Anatomic Reservoir on Low-Flow Oxygen Delivery Via Nasal Cannula: Constant Flow Versus Pulse Flow With Portable Oxygen Concentrator. Respir. Care.

[28] Baboi L., Subtil F., Guérin C. (2016). A bench evaluation of fraction of oxygen in air delivery and tidal volume accuracy in home care ventilators available for hospital use. J. Thorac. Dis..

[29] Hsu W.-C., Orr J., Lin S.-P., Yu L., Tsou M.-Y., Westenskow D.R., Ting C.-K. (2017). Efficiency of oxygen delivery through different oxygen entrainment devices during sedation under low oxygen flow rate: A bench study. J. Clin. Monit..

[30] Arnal J.-M., Garnero A., Saoli M., Chatburn R.L. (2018). Parameters for Simulation of Adult Subjects During Mechanical Ventilation. Respir. Care.

[31] Ward J.J. (2012). High-Flow Oxygen Administration by Nasal Cannula for Adult and Perinatal Patients. Respir. Care.

[32] Wagstaff T.A.J., Soni N. (2007). Performance of six types of oxygen delivery devices at varying respiratory rates*. Anaesthesia.

[33] Chikata Y., Onodera M., Oto J., Nishimura M. (2017). FiO2 in an adult model simulating high-flow nasal cannula therapy. Respir. Care.

[34] Caille V., Ehrmann S., Boissinot E., Perrotin D., Diot P., Dequin P.-F. (2009). Influence of Jet Nebulization and Oxygen Delivery on the Fraction of Inspired Oxygen: An Experimental Model. J. Aerosol Med. Pulm. Drug Deliv..

[35] Markovitz G.H., Colthurst J., Storer T.W., Cooper C.B. (2010). Effective inspired oxygen concentration measured via transtracheal and oral gas analysis. Respir. Care.

[36] O’Reilly-Nugent A., Kelly P.T., Stanton J., Swanney M.P., Graham B., Beckert L. (2014). Measurement of oxygen concentration delivered via nasal cannulae by tracheal sampling. Respirology.

